# *Ralstonia mannitolilytica* sepsis after elective cesarean delivery: a case report

**DOI:** 10.1186/s12884-021-04214-5

**Published:** 2021-10-30

**Authors:** Shenglan Zhou, Dongmei Tang, Sumei Wei, Zhengchang Hu, Xuemei Wang, Dan Luo

**Affiliations:** 1 Department of Critical Obstetrics, Chengdu Women’s and Children’s Central Hospital, School of Medicine, University of Electronic Science and Technology of China, Chengdu, Sichuan Province China; 2 Department of Intensive Care Unit, Chengdu Women’s and Children’s Central Hospital, School of Medicine, University of Electronic Science and Technology of China, Chengdu, Sichuan Province China; 3 Department of Clinical Laboratory, Chengdu Women’s and Children’s Central Hospital, School of Medicine, University of Electronic Science and Technology of China, Chengdu, Sichuan Province China

**Keywords:** *Ralstonia mannitolilytica*, Elective cesarean delivery, sepsis, Septic shock, Case report

## Abstract

**Background:**

*Ralstonia mannitolilytica*, a newly emerging opportunistic pathogen worldwide, has been reported to be responsible for human pneumonia, septicemia and meningitis. This is the first report of a case of *Ralstonia mannitolilytica* sepsis after elective cesarean delivery.

**Case presentation:**

A 25-year-old woman, gravida 1 para 0, was scheduled for an elective cesarean delivery at 39^+ 1^ weeks of gestation. Sudden high fever and decreased blood pressure occurred a short time after the operation. *Ralstonia mannitolilytica* was identified in her blood culture 5 days after the operation. Based on the presence of sepsis and septic shock, massive fluid replacement, blood transfusion, vasoactive agents, imipenem/cilastatin and cefoperazone sulbactam sodium were applied. She was discharged after intensive care without complications.

**Conclusions:**

Although the incidence of sepsis due to *Ralstonia mannitolilytica* is relatively low, once infection occurs in a puerpera, severe symptoms develop abruptly. Thus, prompt diagnosis and appropriate treatment are key to the cure.

## Background

*Ralstonia mannitolilytica* (*R. mannitolilytica*) belongs to the genus Ralstonia of the Burkholderiaceae family and was first isolated by Yabuuchi in 1995 [1]. It exists mainly in different types of water sources and can survive in low-nutrient environments. In recent years, there have been only a few reports on *R. mannitolilytica* infection causing pneumonia, septicemia and meningitis [2]; however, reports on this pathogen are few. Herein, we report the first case of sepsis due to *R. mannitolilytica* in an elective cesarean delivery patient.

## Case presentation

A 25-year-old woman, gravida 1 para 0 with satisfactory antenatal progress was admitted for an elective cesarean delivery by maternal request at 39^+ 1^ weeks of gestation. She denied any medical history of chronic disease. As requested by the patient, combined spinal-epidural anesthesia was administered for cesarean delivery. Fifteen milligrams of 0.5% ropivacainehydro chloride injection (AstraZeneca AB, Sweden) was injected into the subarachnoid space and 5 mL of 2% lidocaine (Shanxi Shiyao Yinhu, China) was injected into the epidural space. Cefathiamidine (1.0 g, Shandong Luoxin, China) was administered intravenously as a prophylactic antibiotic. During the surgery, the patient started shivering, but the vital signs remained stable. The operation was completed successfully, and she was monitored in an anesthetic resuscitation room.

In the anesthetic resuscitation room, she continued to shiver and complained of general numbness and breathlessness. She was febrile with a temperature of 40.7 °C. Her blood pressure dropped to its lowest point of 78/44 mmHg, she had tachycardia with a maximum heart rate of 177 bpm, and she had intermittent vaginal bleeding. However, her oxygen saturation remained at 98%. Hence, septic shock was suspected, but we were unable to rule out anaphylactic shock. Resuscitation was initiated with massive fluid replacement, intravenous dexamethasone (Sinopharm, China), and a uterotonic agent, namely, 250 μg of intramuscular carboprost tromethamine (Pharmacia and Upjohn company, America). Concurrently, a Bakri Postpartum Balloon (Cook, America) was placed. Despite these treatments, her blood pressure remained unstable (< 90/60 mmHg). The blood investigations revealed metabolic acidosis (a pH of 7.252 [7.35–7.45], a serum lactic acid concentration of 6.52 mmol/L [0.36–1.25]), a raised procalcitonin of 94.5 ng/mL [0.0–0.5], an increased white blood cell count of 15 × 10^9^/L [4–10 × 10^9^], increased neutrophilia of 96.1% [50–70%], decreased platelets of 83 × 10^9^/L [100–450 × 10^9^], a hemoglobin concentration of 105 g/L [110–155], a hematocrit of 30.7% [35–45%], coagulopathy with a prolonged activated partial thromboplastin time (APTT) of 91.3 s [22.3–32.5] with low fibrinogen (FIB) < 0.25 G/L[2-4], and hypokalemia of 3.26 mmol/L [3.5–5.5]. The sequential organ failure assessment (SOFA) score was above 2 (oxygenation index ≤400 mmHg, platelet count≤150,000/uL and mean arterial pressure < 70 mmHg). A central venous catheter and a femoral artery catheter were inserted, red blood cell concentrates and fresh frozen plasma were transfused, low-dose noradrenalin (Grandpharma, China) was administered, and the antibiotic was changed to 2.0 g of ceftriaxone sulbactam sodium (Pfizer, America) at 12-h intervals. Five hours after surgery, all vital signs except her body temperature stabilized. Eighteen hours after surgery, her temperature decreased to 37.8 °C, with a raised white blood cell count of 23.13 × 10^9^/L, neutrophilia of 91.7%, procalcitonin above 100 ng/mL, a decreased platelet count of 64 × 10^9^/L, hemoglobin of 71 g/L and hematocrit of 19.8%. The antibiotic dose was altered to 500 mg of intravenous imipenem/cilastatin sodium (Merck Sharp &Dohme, America) at 6-h intervals. Five days after surgery, the results of the blood culture and microbe identification confirmed *R. mannitolilytica*. At that time, the patient’s body temperature had fluctuated to approximately 37.4 °C, and the hemogram decreased significantly (white blood cell count, 10.43 × 10^9^/L; neutrophilia, 76.9%; procalcitonin, 11.6 ng/mL). Treatment with imipenem/cilastatin sodium was continued because the drug sensitivity test of the bacteria could not be carried out in our laboratory. Eight days after surgery, the patient’s body temperature returned to normal. Blood analysis showed a white blood cell count of 12.09 × 10^9^/L, neutrophilia of 76.6% and procalcitonin of 2.26 ng/mL. The antibiotic was changed to 2.0 g of intravenous cefoperazone sulbactam sodium at 6-h intervals for 6 days. All the blood results had returned to normal, the repeated blood culture was negative on day 14 postoperatively and she was discharged well on day 15. No abnormality was found during follow-up at half a year after discharge.

## Discussion and conclusions

*R. mannitolilytica* was first reported in 1995, and since then, there have been case reports on infections involving neonatal, oncological, dialysis and renal transplantation patients [3–7]. In 2011, *R. mannitolilytica* was first isolated in China from a patient with chronic obstructive pulmonary disease [8]. *R. mannitolilytica* infects humans mainly through water sources and affects mainly immunocompromised groups [3–8]. The common sources of contamination in hospitals are water used during dialysis, sterile water for injection, and oxygen delivery systems [3, 5, 7]. However, in most cases, the source is unknown. In our case, pregnancy was associated with a transient immunocompromised state, which put her at higher risk for *R. mannitolilytica* infection. However, the source of the infection is still unknown. She had rapid progression to critical septic shock, and there were no subsequent cases in our hospital.

Obstetric sepsis is among the top 3 leading causes of maternal death worldwide, and can lead to multiple organ or system dysfunction and even failure [9]. In most reported cases, *Escherichia coli*, Streptococcus and Staphylococci are the causal bacteria of severe obstetrical infectious events [10]. This is the first report of maternal infection caused by *R. mannitolilytica*, and this case confirms the notion that this opportunistic pathogen causes maternal infections. Due to the diversity of infected tissues and organs, the main clinical manifestations of *R. mannitolilytica* infection are pneumonia, bacteremia, and meningitis, and a few cases develop septic shock. The main symptoms of the current case were fever and hypotension, which progressed to septic shock a short time after the operation. These symptoms were basically consistent with descriptions in the literature [4, 11, 12].

When the pathogen was not clear, initial treatment with supportive therapy and an empirical broad-spectrum antibiotic strategy could be selected according to the American Critical Medical Association’s 2016 International guidelines for Treatment of Sepsis and Septic Shock [13]. Concurrently, pathogen culture and antimicrobial susceptibility testing were carried out. *R. mannitolilytica* can produce various enzymes that can hydrolyze antibiotics. These compounds can confer resistance to a broad range of antibiotics, including benzylpenicillin, narrow-spectrum cephalosporins, ceftazidime, aztreonam, and carbapenems [14]. Additionally, different strains show different drug resistance [2]. Daxboeck et al. reported that among the 30 strains isolated, 12 strains developed resistance to carbapenem antibiotics [12]. In Said’s studies, all strains were resistant to meropenem but sensitive to imipenem [6]. Due to technical restrictions and equipment constraints, antimicrobial susceptibility testing was not carried out. Imipenem cilastatin sodium was used in our patient according to reported experience and clinical practice.

After treatment with antibiotics, most patients, though not those with cystic fibrosis, who are infected with *R. mannitolilytica* infection have a good prognosis [15]. However, there is no unified standard for the treatment time. Procalcitonin kinetics have been proven to have prognostic value correlating with disease severity and resolution of illness. For more severe infections (such as sepsis), determining antibiotic treatment by monitoring procalcitonin kinetics resulted in a shorter antibiotic treatment duration with early cessation of therapy. These strategies appear to be safe without increasing the risk of mortality, recurrent infections or treatment failure [16]. According to the experience of this case, when the clinical manifestations and procalcitonin level were significantly abnormal, antibiotics were used. Once procalcitonin dropped to levels < 0.5 ng/ml in combination with clinical improvement, the treatment was stopped, and this resulted not only in satisfactory results but also in no related complications.

In summary, *R. mannitolilytica*, a new opportunistic pathogen, can contribute to critical infectious events. As a special patient group, pregnant and postpartum women should be assessed for the possibility of infection with this pathogen if rapid sepsis and septic shock occur. Early diagnosis and appropriate antibiotics can decrease complications.

## Data Availability

All data generated or analyzed during this study are included in this published article.

## Abbreviations

*R. mannitolilytica*: *Ralstonia mannitolilytica*
APTT: Activated partial thromboplastin time
FIB: Fibrinogen
SOFA: Sequential organ failure assessment

## References

[1] De Baere T, Steyaert S, Wauters G (2001). Classification of Ralstonia pickettii biovar 3/'thomasii' strains (Pickett 1994) and of new isolates related to nosocomial recurrent meningitis as Ralstonia mannitolytica sp. nov. Int J Syst Evol Microbiol.

[2] Ryan MP, Adley CC (2014). Ralstonia spp.: emerging global opportunistic pathogens. Eur J Clin Microbiol Infect Dis.

[3] Jhung MA, Sunenshine RH, Noble-Wang J (2007). A national outbreak of Ralstonia mannitolilytica associated with use of a contaminated oxygen-delivery device among pediatric patients. Pediatrics..

[4] Gröbner S, Heeg P, Autenrieth IB, Schulte B (2007). Monoclonal outbreak of catheter-related bacteraemia by Ralstonia mannitolilytica on two haemato-oncology wards. J Inf Secur.

[5] Lucarelli C, Di Domenico EG, Toma L (2017). Ralstonia mannitolilytica infections in an oncologic day ward: description of a cluster among high-risk patients. Antimicrob Resist Infect Control.

[6] Said M, van Hougenhouck-Tulleken W, Naidoo R (2020). Outbreak of Ralstonia mannitolilytica bacteraemia in patients undergoing haemodialysis at a tertiary hospital in Pretoria, South Africa. Antimicrob Resist Infect Control.

[7] Shankar M, Rampure S, Siddini V (2018). Outbreak of Ralstonia mannitolilytica in hemodialysis unit: a case series. Indian J Nephrol.

[8] Zong ZY, Peng CH (2011). Ralstonia mannitolilytica and COPD: a case report. Eur Respir J.

[9] Chebbo A, Tan S, Kassis C (2016). Maternal Sepsis and septic shock. Crit Care Clin.

[10] Acosta CD, Kurinczuk JJ, Lucas DN (2014). Severe maternal sepsis in the UK, 2011-2012: a national case-control study. PLoS Med.

[11] Liu CX, Yan C, Zhang P (2016). Ralstonia mannitolilytica-induced septicemia and homology analysis in infected patients: 3 case reports. Jundishapur J Microbiol.

[12] Daxboeck F, Stadler M, Assadian O (2005). Characterization of clinically isolated Ralstonia mannitolilytica strains using random amplification of polymorphic DNA (RAPD) typing and antimicrobial sensitivity, and comparison of the classification efficacy of phenotypic and genotypic assays. J Med Microbiol.

[13] Rhodes A, Evans LE, Alhazzani W (2017). Surviving Sepsis campaign: international guidelines for Management of Sepsis and Septic Shock: 2016. Intensive Care Med.

[14] Prior AR, Gunaratnam C, Humphreys H (2017). Ralstonia species - do these bacteria matter in cystic fibrosis?. Paediatr Respir Rev.

[15] Boattini M, Bianco G, Biancone L, Cavallo R, Costa C (2018). Ralstonia mannitolilytica bacteraemia: a case report and literature review. Infez Med.

[16] Sager R, Kutz A, Mueller B (2017). Procalcitonin-guided diagnosis and antibiotic stewardship revisited. BMC Med.

